# Spinal extradural angiolipomas: A case report

**DOI:** 10.1016/j.amsu.2021.102289

**Published:** 2021-04-13

**Authors:** Othman Benhayoun, Marouane Makhchoune, Abdelhamid Jehri, Mohamed Yassine Haouas, Said Hilmani, Abdelhakim Lakhdar

**Affiliations:** Neurosurgery Department, University Hospital Center IBN ROCHD, Casablanca, Morocco

**Keywords:** Spinal extradural, Angiolipomas, MRI, Case report

## Abstract

Angiolipoma are benign tumors composed of mature adipocytes ad mixed with abnormal vascular elements. It reportedly accounts for 0.1–0.5% of all spinal axis tumors in adults and is extremely rare in children.

This article describes a case of spinal extradural angiolipoma in a 35-year-old man, who presented with paraplegia and sphincter disorders, the MRI showed extradural spinal lesions at the level of T7 to T10, that squeezed the spinal cord. We attained total resection without any further neurological complication.

The diagnosis of SEALs initially can be challenging radiologically since they may imitate other spinal lesions. The gold standard treatment modality should always be surgery, however in some cases, total resection can not be done.

## Highlights

• This paper presents a 35-year-old man complaining for 1 months a numbness and lowerlimbs heaviness. The neurological examination revealed paraplegia with a muscle power was 2/5. However, in lower limbs, the deep tendon reflexes were absent, walking was impossible also without any sensitive disorder.
• MRI revealed T7-T10 extradural spinal lesion. The tumor appeared as an hyperintense on T1 and T2-weighted images, and as heterogeneous on STIR. Total resection after dissecting the tissue around the tumor. The histopathological examination of the specimens obtained, showed that the lesion was made up of fatty tissue and angioma-like tissue.
• SAL is a rare benign tumor and considered as a specific clinico-pathological entity. MRI is considered to be the “gold standard” in diagnosing SAL and surgery appears to be the treatment of choice.

## 1 Introduction

Angiolipomas are benign tumors that probably originate from the same progenitor tissue as lipomas and hemangiomas. They may represent an intermediate stage between the two extremes [{1}]. They are composed predominantly of vascular and stromal elements, with lipomatous component and blood vessels. Spinal angiolipomas are a specific clinico-pathological entity, but extremely uncommon. They are reported for 0.04–1.2% of all spinal axis tumors in adults and 2–3% of extradural spinal tumors [[1], [2]].

Spinal angiolipomas affect predominantly women [{3}]. Mostly, they involve in the thoracic region, and generate symptoms and signs of spinal compression. In some cases, they can erod bones and cause pathological fractures [{4}]. Their symptomatology can be exacerbated by pregnancy and weight gain, suggesting that vascular engorgement and the presence of obesity influence their evolution [{5}]. Their preponderance in older, peri-menopausal, or postmenopausal women, and their clinical exacerbation in pregnant women support a role for hormonal influence [{6}].

Given their rarity, they are often overlooked in the differential diagnosis of space-occupying lesions within the spinal canal. Mostly, Spinal angiolipomasdo not infiltrate, but in some cases, an infiltration into the surrounding soft tissues and vertebrae may occur, accordingly, it can mimic a malignant tumor [[7], [8]]. Magnetic resonance imaging is the investigation of choice for the diagnosis of these lesions. Surgery is universally successful in relieving symptoms. Currently, there is no universal consensus on treatment. Surgery advocating total resection is the main treatment which may be an aggressive option in some cases [[9], [10]].

We report one case of spinal angiolipoma within the neurosurgery department at the Ibn Rochd university hospital in Casablanca.

## 2 Case report

We present a case of 35-year-old man without history suggestive of any mental or physical illness, brought by ambulance with his wife, he presented to our emergency department with the chief complains of numbness and lowerlimbs heaviness for 1 months. Theses symptoms progressed very gradually until he could no longer walk and he experienced difficulty in passing urine as well as fecal incontinence.

On examination, find paraplegia with a muscle power was 2/5. The muscle bulk, power and reflexes on the upper limbs were normal. However, in lower limbs, The deep tendon reflexes were absent, Babinski responses were positive and walking was impossible also without any sensitive disorder.

**Fig. 1:**
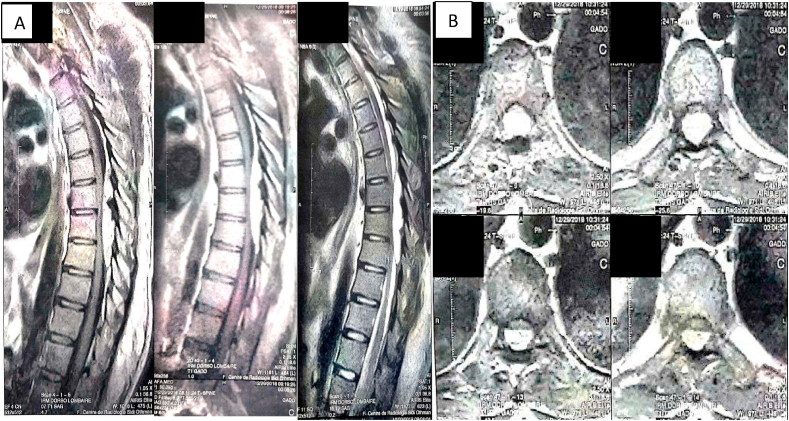
(A,B): Post-contrast T1-weighted sagittal MRI (A) showing a homogeneous hyperintense mass at T7-T10, axial MRI (B) compressing the spinal cord obvious enhancement of the mass.

MRI revealed T7-T10 extradural spinal lesion (Fig. 1). The lesion measured about 8.0 cm × 1.2 cm and located at the dorsal spinal cord. The tumor appeared as an hyperintense on T1 and T2-weighted images, and as heterogeneous on STIR.

The lesion showed maximal enhancement after intravenous (IV) contrast administration and exerted compressive effect on the spinal cord.

We thought of meningioma, metastasis but he was in good shape and cancer has been reported, chronic extradural hematoma but there was no traumatic context.

After education and counselling of the patient and his familly, surgery was scheduled. The intervention was performed by our professor with 22 years of experience under general anesthesia. The goal of the surgery was to decompress T7–T10 spinal nerve root via T7–T10 laminotomy. Intraoperatively, we found the lesion at the T7– T10 dorsal spinal cord. The lesion was at the epidural space. It measured about 8 cm × 1.8 cm. The lesion was red in color and tough in consistence with regular margins. It had very rich blood supply. We achieved total resection after dissecting the tissue around the tumor without any incident.

The histopathological examination of the specimens obtained, showed that the lesion was made up of fatty tissue and angioma-like tissue. The follow up remains stationary paraplegia with a muscle power was 2/5, he was discharged from the hospital on the 5th day following the intervention and send for kinesitherapy Because of severe preoperative neurological deterioration of our patient, a year and a half follow-up period has revealed no signs of tumor recurrence but no recovery of his initial neurological deficit.

This case has been reported in line with the 2020 SCARE guidelines [{11}].

## 3 Discussion

Angiolipomas are an uncommon form of lipomas, they are benign tumor of fatty tissue and differs from lipomas because of the presence of more blood vessels running through them. They was described as a distinct anatomopathologic entity by howard et al., in 1960 [{12}]. Spinal angiolipomas (SALs) represent less than 2% of all spinal axis tumors. They are categorized into two subtypes: Non-infiltrating and infiltrating types. The non-infiltrating types are more common and usually well encapsulated. The infiltrating types are rare, partially or entirely unencapsulated and they have a locally aggressive nature. SALs can erode the local bone, muscle, and neural and fibrocollagenous tissues [{13}].

Most angiolipomas occur mostly in adults aged between 40 and 60 years with a few cases observed in pediatric patients. The age of our patient (35 years-old) is consistent with the average ages of patients initially reported in the literature [[14], [15]]. In contrast to our case, a female predominance has been observed in the series reported in the littérature [{16}]. The most common location of spinal angiolipoma is the thoracic epidural space. Thoracic predominance of the tumor could be the result of the regional variation in the blood supply in the spine, where in the mid-thoracic spine is the least perfused [{16}]. In our patient, the tumor was located in the T7-T10 dorsal spinal cord in the epidural space.

SALs appear to be slow growing, the anatomical location of these tumors predisposes them to produce early spinal cord compression due to the limited space available in the spinal canal. The frequent clinical presentations are progressive paraparesis, back pain, lower extremity sensory changes, and hyper reflexia. The symptoms usually evolve over a span of months to years [{16}]. Some times, the symptoms onset and exacerbations have been reported during pregnancy, probably because of impaired spinal venous drainage or hormonal changes [{17}]. In our case as described in differentes series, presented with 1 month history of numbness and lower limbs heaviness. Then symptoms progressed verygradually until He could no longer walk. Diagnosis of SEAL was suspected on clinical examination and MRI images then confirmed by histology after surgery. The magnetic resonance imaging is considered to be the “gold standard” in diagnosing SAL. In our case, the magnetic resonance image indicated the possibility of a vascularized lipid-containing tumor on T7-T10. In general, on T1-weighted images, SAL are hyperintense and often in homogeneous with variable intensity. They are often hyperintense on T2-weighted images. Significant heterogeneity in the imaging studies is attributed to the variable vascular and adipose elements of the tumor [{18}]. Additionally, spinal lipomas occur most commonly in the midline of the lower cervical, upper thoracic, and lumbar spine regions that correspond to the last sites of closure of the embryonic neural arch and are associated with dysraphic anomalies of the neuraxis [{19}].

The differential diagnosis includes extradural lipomatosis, nerve sheath tumor, meningioma, metastasis, chronic extradural hematoma, and lymphoma. An easy misdiagnosisis extradural lipomatosis, in which, The lipoma is always accompanied by spina bifida, and usually seen among the youngest patients, and more common in men especially in the lumbosacral region [{20}].

Surgery appears to be the treatment of choice. In most cases complete excision appears to be curative. Especially for non-infiltrating SAL dorsally located, as they are generally located in the posterior portion of the epidural space and well delimited from the surrounding tissue, they can usually be removed easily by laminectomy. For the infiltrating type, a total resection can be more difficult. However, most patients have a good prognosis even with subtotal removal, according to the littérature, as these lesions are slowly growing and do not undergo malignant transformation [{21}].

## 4 Conclusion

In summary, SAL is a rare benign tumor and considered as a specific clinico-pathological entity, composed of varying proportions of mature fat cells and vascular elements. They often occur in middle-aged female patients and generally located in the dorsal epidural space of the thoracic spine. MRI is considered to be the “gold standard” in diagnosing SAL and surgery appears to be the treatment of choice with good outcomes.

## Research registration unique identifying number (UIN)

Please enter the name of the registry and the unique identifying number of the study. You can register your research at http://www.researchregistry.com to obtain your UIN if you have not already registered your study. This is mandatory for human studies only.

None.

## If you are submitting an RCT, please state the trial registry number – ISRCTN

None.

## Author contribution

Please specify the contribution of each author to the paper, e.g. study design, data collections, data analysis, writing. Others, who have contributed in other ways should be listed as contributors.

Othman BENHAYOUN: writing the paper.

Marouane MAKHCHOUNE: Corresponding author and writing the paper.

Adelhamid JEHRI: study concept.

Mohamed yassine HAOUAS: Correcting the paper.

Said HILMANI: Correcting the paper.

Abdelhakim LAKHDAR: Correcting the paper.

## Guarantor

The Guarantor is the one or more people who accept full responsibility for the work and/or the conduct of the study, had access to the data, and controlled the decision to publish.

MAKHCHOUNE MAROUANE.

## Financial disclosure

The authors declared that this study has received no financial support.

## Ethical approval

Written informed consent was obtained from the patient for publication of this case report and accompanying images. A copy of the written consent is available for review by the Editor-in-Chief of this journal on request.

## Provenance and peer review

Provenance and peer review Not commissioned, externally peer-reviewed.

## Declaration of competing interest

The authors of this article have no conflict or competing interests. All of the authors approved the final version of the manuscript.
